# OCT Hyperreflective Retinal Foci in Diabetic Retinopathy: A Semi-Automatic Detection Comparative Study

**DOI:** 10.3389/fimmu.2021.613051

**Published:** 2021-04-22

**Authors:** Edoardo Midena, Tommaso Torresin, Erika Velotta, Elisabetta Pilotto, Raffaele Parrozzani, Luisa Frizziero

**Affiliations:** ^1^ Department of Ophthalmology, University of Padova, Padova, Italy; ^2^ IRCCS—Fondazione Bietti, Rome, Italy

**Keywords:** inflammation, OCT, hyperreflective retinal foci, diabetic retinopathy, automatic detection, biomarker

## Abstract

Optical coherence tomography (OCT) allows us to identify, into retinal layers, new morphological entities, which can be considered clinical biomarkers of retinal diseases. According to the literature, solitary, small (<30 µm), medium level hyperreflective (similar to retinal fiber layer) retinal foci (HRF) may represent aggregates of activated microglial cells and an *in vivo* biomarker of retinal inflammation. The identification and quantification of this imaging biomarker allows for estimating the level and possibly the amount of intraretinal inflammation in major degenerative retinal disorders, whose inflammatory component has already been demonstrated (diabetic retinopathy, age-related macular degeneration, radiation retinopathy). Currently, diabetic retinopathy (DR) probably represents the best clinical model to apply this analysis in the definition of this clinical biomarker. However, the main limitation to the clinical use of HRF is related to the technical difficulty of counting them: a time-consuming methodology, which also needs trained examiners. To contribute to solve this limitation, we developed and validated a new method for the semi-automatic detection of HRF in OCT scans. OCT scans of patients affected by DR, were analyzed. HRF were manually counted in High Resolution spectral domain OCT images. Then, the same OCT scans underwent semi-automatic HRF counting, using an ImageJ software with four different settings profiles. Statistical analysis showed an excellent intraclass correlation coefficient (ICC) between the manual count and each of the four semi-automated methods. The use of the second setting profile allows to obtain at the Bland–Altman graph a *bias* of −0.2 foci and a limit of agreement of ±16.3 foci. This validation approach opens the way not only to the reliable and daily clinical applicable quantification of HRF, but also to a better knowledge of the inflammatory component—including its progression and regression changes—of diabetic retinopathy.

## Introduction

With the advent of new retinal imaging techniques, such as spectral domain optical coherence tomography (SD-OCT), new findings across the retinal layers, such as hyperreflective intraretinal foci (HRF) have been detected. HRF are described as small, punctiform hyperreflective lesions, visible on linear OCT scans in the retina, either in normal subjects and in patients with signs of chorio-retinal diseases (1–8). They were described for the first time by Coscas et al. (1) in age-related macular degeneration, and proposed as possible marker of activated microglia cells. However, their origin continues to be under debate. Bolz et al. (2) were the first to describe the presence of hyperreflective retina foci in patients with diabetic macular edema (DME), but they were considered to correspond to extravasated lipoproteins and/or proteins. Recently, hyperreflective retinal foci have been identified as a “family” of OCT hyperreflective lesions, which may be differentiated according to their OCT features, namely size, retinal location and reflectivity level. In particular, solitary, small (<30 µm), medium level hyperreflective (similar to the reflectivity of the retinal nerve fiber layer (RNFL)) foci, at least initially mostly located in the inner retina were proved not to correspond to any specific lesion on color fundus images and enface OCT scans, such as vessels and exudates. These HRF have been proposed to represent activated, aggregated, proliferating microglial cells, and, thus, an *in vivo* biomarkers of local inflammation (4, 6, 7, 9, 10). The term HRF will be used in this manuscript as synonymous of this kind of hyperreflective intraretinal foci. HRF—as we defined them—have been particularly studied in diabetic retinopathy, and may allow, together with other biomarkers, to identify a specific pattern/phenotype of DR and DME, prevalently inflammatory, that may differently respond to treatments (11).

One of the main issues in the clinical application of HRF is that that their manual counting on OCT scans depends on the learning curve of the examiner, and it takes time to be reliable and reproducible. For this reason, it can be understood the importance of a new approach able to carry out the HRF count in a semi-automated, standardized, reproducible, fast and objective way.

In this study we report the experience of the development of a method that allows semi-automatic HRF count, validating it versus the manual reference count made by an expert operator.

## Materials and Methods

This is a retrospective evaluation of OCT images obtained in eyes affected by DR and collected from February to June 2020 (during COVID pandemic). This research was carried out in accordance with the Declaration of Helsinki. Because images were completely anonymous, a communication to the Local Review Board was performed, according to the current Italian and Regional legislation. A horizontal 180° linear OCT scan of 8.8 mm centered onto the fovea acquired in High Resolution modality with a 100 ART resolution was carried out using Spectralis HRA + OCT (Heidelberg Engineering, Heidelberg, Germany; Software 6.0.10.0).

**Figure 1:**
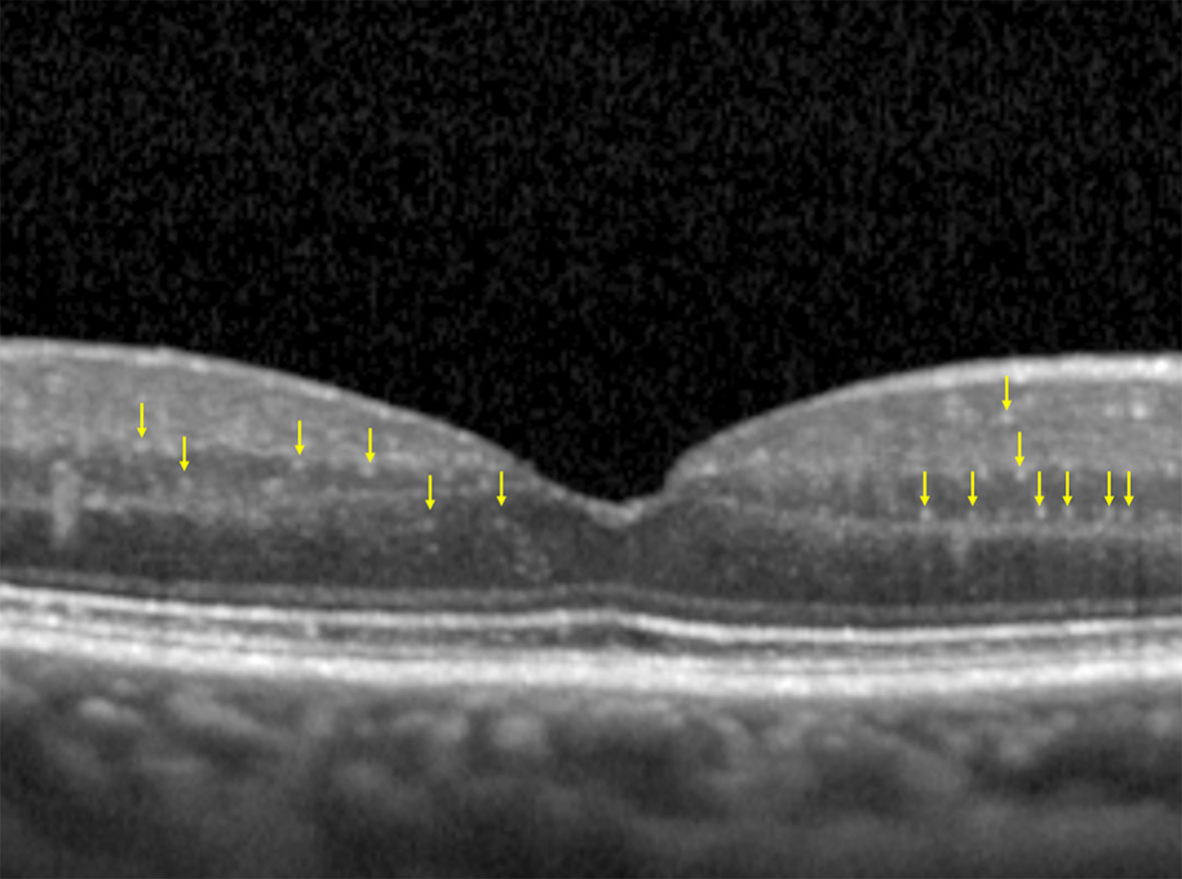
Cropped OCT scan, highlighting central 3 mm, with an example of HRF manually identified (yellow arrows). Note the absence of back-shadowing, the dimension <30 µm and the moderate reflectivity.

Inclusion criteria for the OCT scans were: correct centering of the scan; image quality >30 (automatic value provided by the instrument). Each OCT scan was cropped at 1,500 µm from the fovea, both on the nasal and temporal side, with the aim of highlighting the central 3 mm of the retina, as previously reported (9, 11) (**Figure 1**).

The final OCT images were anonymized and then examined, in a blind fashion, by an expert operator who performed a manual count of the HRF, as defined according to the following characteristics: diameter <30 µm, absence of back-shadowing, and reflectivity similar to that of RNFL.

**Figure 2:**
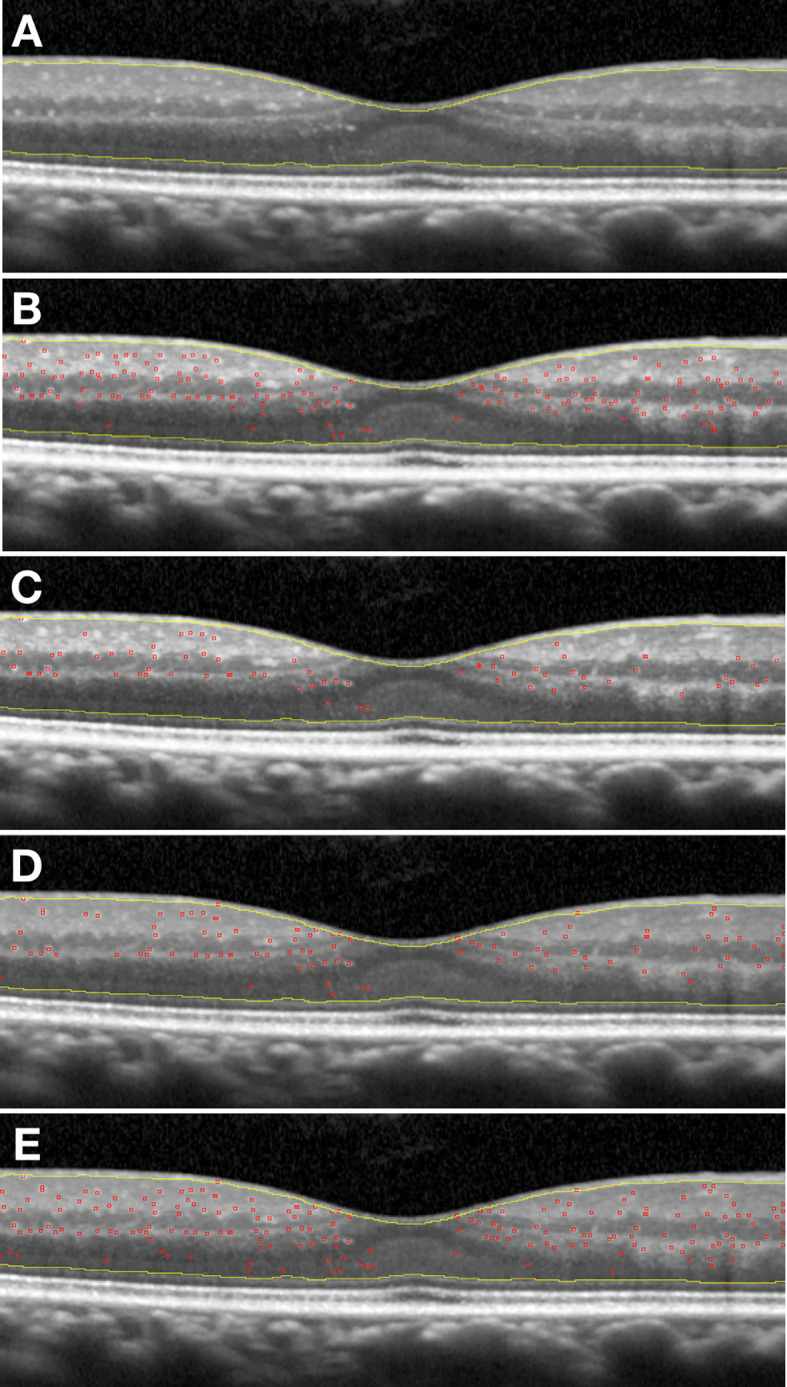
**(A)** Original scan cropped, with region of interest free-hand selected; **(B)** Example of application First semi-automatic method; **(C)** Example of application Second semi-automatic method; **(D)** Example of application Third semi-automatic method; **(E)** Example of application Fourth semi-automatic method.

HRF were counted from the boundary between RNFL and ganglion cell layer (GCL) to the external limiting membrane (ELM). The reflectivity of RNFL, in fact, is too similar to that of the HRF to allow for any reliable distinction. Similarly, in retinal layers external to the ELM, any photoreceptor debris or pigmentary material may confound the exact HRF count. Subsequently, the same OCT images underwent a semi-automated analysis using an open-source available ImageJ software (National Institutes of Health, Bethesda, Maryland, USA). Four different profiles of settings were applied to the software for the analysis of each image and the results were then compared to those obtained with the manual count. Briefly, the cropped images were imported in the software and the 8-bit format was settled. For the first and second method the plugin “*A_trous_filter.java* filter” (coefficient values: k1: 4.0; k2: 3.0; k3: 3.0; k4: 0.0; k5: 0.0; *Non gaussian noise removal* option checked; standard deviation value: 1.50) was applied. The “denoised image” obtained was used as mask for applying the *CLAHE* (Enhance Local Contrast) tool setting (Blocksize: 127; Histogram bins: 256; Maximum slope: 3.00). On the resulting image the *Spot Counter* plugin was applied with different setting for each of the four methods (**Table 1**) on the region of interest from the boundary between RNFL and GCL and ELM identified using the *Freehand selection function*. The following obtained values were then saved into a database: (a) n (whole number of spots counted); (b) spot mean (average value of spot intensity); (c) image mean (average value of image intensity); (d) list of each single spot intensity. The semi-automated analysis procedure is fully described in **Table 1** and an example of the result of each method is reported in **Figure 2**.

**Table 1:** Semi-automated analysis procedure.

	First method	Second method	Third method	Fourth method
**STEP 1**	Import the OCT scan into ImageJ			
**STEP 2**	Set 8-bit format			
**STEP 3**	Run *A-trous-filter.java*: k1: 4.0; k2: 3.0; k3: 3.0; k4: 0.0; k5: 0.0 Non gaussian noise removal **✓** Std dev: 1.50			
**STEP 4**	A Denoised image is obtained			
**STEP 5**	Select main image			
**STEP 6**	Process			
**STEP 7**	*CLAHE* (Enhance Local Contrast): Blocksize: 127 Histogram bins: 256 Maximum slope: 3.00 Mask: denoised image			
**STEP 8**	Plugin			
**STEP 9**	Analyze			
**STEP 10**	*Spot Counter*: Pre-filter: Gaussian_1.5 Box Size: 2 Noise Tolerance: 15	*Spot Counter*: Pre-filter: Gaussian_1.5 Box Size: 2 Noise Tolerance: 20	*Spot Counter*: Pre-filter: None Box Size: 2 Noise Tolerance: 20	*Spot Counter*: Pre-filter: None Box Size: 2 Noise Tolerance: 15
**STEP 11**	*Free hand selection:*			
	• Select the area from RNFL/GCL to ELM			
**STEP 12**	Confirm the *Spot Counter* command			
**STEP 13**	Copy the following values into a database:			
	• n (whole number of spots counted)			
	• spot mean (average value of spot intensity)			
	• image mean (average value of image intensity)			
	• list of each single spot intensity.			

The settled parameters have been specifically designed to prevent the software from counting any hard exudates or retinal vessels as HRF. However, a further correction factor was included to avoid the influence of the average intensity of the entire OCT image on HRF count. Then, the average image intensity value was multiplied by 2 and all HRF with an intensity greater than this value were excluded. This allowed for a greater precision in counting only those foci that really represent aggregates of activated microglia.

### Statistical Analysis

The ability of the method to reproduce the manual count was assessed using ICC and the Bland–Altman diagram (12). The following characteristics of the points cloud are assessed: (a) proximity to zero of the mean value of the differences; (b) amplitude of the range of agreement respect to the order of magnitude of the counts carried out; (c) absence of trend; (d) homogeneity of differences variability on varying of count mean values. Inter-modality reproducibility was performed for the four methods.

## Results

We collected 194 OCT linear scans of 97 diabetic patients. Forty-eight scans were excluded because of image quality <30, and six additional scans were excluded because of uncorrected centering of the scan. Thus, 140 OCT linear scans of 89 diabetic patients were processed. The mean age was 65.8 ± 9.5 years: 40 were female and 49 were male. Five patients were affected by type 1 diabetes (4.5%) and 84 by type 2 diabetes. All patients were affected by non-proliferative DR.

**Figure 3:**
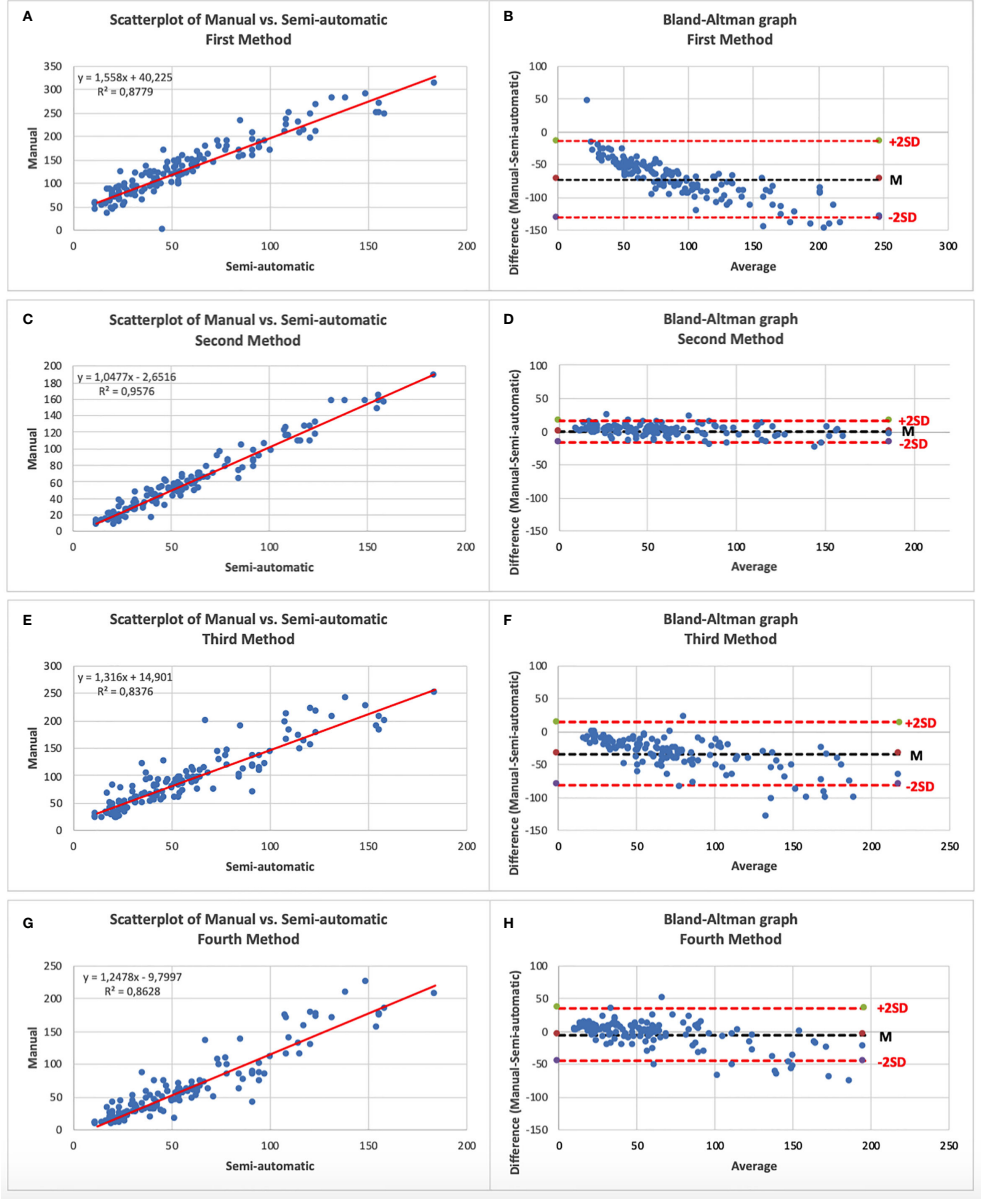
**(A)** Scatterplot of Manual vs. First semi-automatic method; **(B)** Bland–Altman graphic of Manual vs. First semi-automatic method; **(C)** Scatterplot of Manual vs. Second semi-automatic method; **(D)** Bland–Altman graphic of Manual vs. Second semi-automatic method; **(E)** Scatterplot of Manual vs. Third semi-automatic method; **(F)** Bland–Altman graphic of Manual vs. Third semi-automatic method; **(G)** Scatterplot of Manual vs. Fourth semi-automatic method; **(H)** Bland–Altman graphic of Manual vs. Fourth semi-automatic method.—An excellent correlation can be noted on all scatterplots. Note on the Bland–Altman graphs the high variability of the differences between the two counting modalities in the methods 1, 3 and 4 compared to the little variability in method 2 (narrower cloud of points).

The mean number of HRF at manual count was 58.9 ± 36.9 foci. After comparison of the four semi-automatic counting method, the second method showed the best results in terms of mean count of HRF (58.6 ± 39.7 foci) compared to the manual counting method, with a ICC of 0.98, a bias of −0.2 foci, and a range of ±16.3 foci at the Bland–Altman diagram (**Figure 3**) The mean count, ICC, the bias and the range of agreement of each semi-automated method are fully reported in **Table 2**.

**Table 2:** Statistical analysis results of each method.

	Count *(n° foci, mean ± SD)*	ICC	Bias* (n° foci)*	Range of agreement *(n° foci)*
**First method**	131.0 ± 62.1	0.94	−73.1	± 58.2
**Second method**	58.6 ± 39.7	0.98	−0.2	± 16.3
**Third method**	91.7 ± 53.4	0.92	−33.5	± 47.7
**Fourth method**	63.19 ± 49.65	0.93	−4.78	± 40.17

## Discussion

The use of objective, quantifiable medical signs, named as biomarkers, has become of greater relevance in clinical practice and research. In fact, they not only allow the identification of a normal biological or pathological process but also the quantification and follow-up of the process itself, becoming clinical and diagnostic tools in the common practice but also surrogate endpoints in clinical research (13). Moreover, in recent years artificial intelligence in medicine and notably in ophthalmology has assumed a growing relevance (14). In fact, the automatic detection of pathology-related features appears as a promising approach in disease treatment and monitoring, but it requires precise characteristics and classification of a specific biomarker, in order to obtain a semi-automated evaluation.

Several steps forward have been made since Coscas et al. (15) first noticed the presence of HRF scattered across all retinal layers, in OCT scans of patients with advanced age-related macular degeneration. Subsequent studies have demonstrated that hyperreflective retinal foci represent a family of OCT hyperreflective retinal elements, among which those with a diameter >30 µm, with back-shadowing and located in the inner retina may be classified as retinal vessels; those >30 µm, with back-shadowing, with reflectivity similar to EPR and mainly located in the outer retina have been identified as hard exudates. As previously reported the term HRF, or solitary HRF, is referred to those defined by a diameter <30 µm, absence of back-shadowing and reflectivity similar to the RNFL. Although their nature is still under discussion, one of the main hypotheses refers these HRF as aggregates of activated microglia (7). Therefore HRF have been proposed as a marker of neuro-retinal inflammation, identifiable by analyzing linear OCT scans. The identification and quantification of this imaging biomarker may allow to identify the inflammatory component in major vascular and degenerative retinal disorders, such as DR and age-related macular degeneration. Ocular proteomics studies, performed on vitreous and aqueous humor of patients with DR (with or without DME) showed the presence of a well-defined inflammatory phenotype, characterized by high levels of VEGF, ICAM1, MCP1, IL6, IFN and IL-1beta, and the correlation of this inflammation with the severity of the disease (16–18). High levels of pro-inflammatory cytokines associated with the accumulation of advanced glycation end-products (AGEs) and advanced lipoxidation end-products (ALEs) were demonstrated to be induced by retinal microglia activation (19, 20). Several studies were focused on the presence of HRF in patients with or without clinically detectable DR (6, 21). HRF proved to increase with the progression of DR, suggesting the close correlation between the presence of these lesions and the degree of retinal inflammation in DR (6). Moreover, correlation between sCd14 levels, a cytokine related to the activation of the inflammatory response, in the human aqueous humor and HRF in the inner retina has been reported (22). It has also been demonstrated that the number of HRF increases in DME associated with subfoveal retinal detachment (SRD), compared to DME without SRD (23). Proteomic studies on aqueous or vitreous samples of eyes with DME and SRD have confirmed an increased concentration of inflammatory molecules, especially interleukin 6, thus reinforcing the presence of a predominant inflammatory pathogenesis in this type of patients (17, 24). Regardless of the nature of HRF, several studies showed a significant and fast decrease of HRF in OCT scans of patients who underwent intravitreal treatment with anti-VEGF drugs (4, 11, 25), or even more with dexamethasone (11).

Moreover, some Authors agree that the presence and number of HRF is a reliable biomarker of retinal response to treatment and even hypothesized an independent predictor of visual outcome, in DME but also in other retinal vascular diseases (26, 27).

The quantification of HRF may be useful not only in DR. Several studies showed the importance of the number and location of HRF, from a diagnostic and prognostic point of view, in diseases such as age-related macular degeneration (1), multiple sclerosis (10) and radiation retinopathy (8). An increase in HRF can be observed even before the onset of macular edema, which highlights how HRF could support the decision of preventive treatments to avoid reaching clinical situations that would require more aggressive therapies (28). The main current issue is that the manual count of HRF on OCT scans requires experience and takes time to be reliable and reproducible. The lack of a semi-automated calculation limits the use of this well recognized imaging biomarker to some more experience centers, preventing the relevant clinical information which may offer. Papers related to the automatic or semi-automatic count of HRF are limited in number and partly unreliable, and theoretically valid only from an informatic and technical point of view (29–31). The method proposed by Marzieh et al. lacks both retinal morphology, and a clinical contribution. This approach seems inapplicable in the clinical setting, since it ignores data regarding the characterization of the different retinal hyperreflective foci on the basis of diameter and degree of reflectivity. Yu et al. defined hyperreflective retinal foci as hard exudates, while Varga et al. incorrectly characterized HRF attributing them a reflectivity similar to that of retinal pigment epithelium, mainly counting just hard exudates. Our approach overcomes these limitations because we developed a method able to identify HRF, as previously defined, excluding hard exudates and vessels. Moreover, we tested the software with four different configuration profiles and the method number 2, which we named “semi-automatic HRF counter”, resulted the most reliable compared to the manual count, which currently represents the gold standard. The method number 2, in fact, was characterized by the higher ICC (0.98), the lower bias (−0.2 foci) and the narrower range of agreement ( ± 16.3 foci). This means that the semi-automatic HRF counter counts an average number of foci comparable to manual counting and that the difference between the two counting modes has a low variability. Although all the other methods are characterized by an excellent ICC (**Table 2**), they have the limit of a high variability of the difference between the semi-automatic and manual counting depending on the average number of HRFs present in the image. Furthermore, the semi-automatic method numbers 1 and 3 also tend to count a number of HRFs on average too high compared to manual counting. Therefore, our validation approach identified, in the second method, a semi-automated approach which can completely and reliably replace the experienced operator in counting HRF. The integration of this validated tool directly into OCT software might help physicians in clinical practice, allowing them to detect and quantify HRF, as a possible biomarker of a specific retinal phenotype in retinal diseases, improving the management of the single patient.

## Conclusions

In conclusion, this study provides a semi-automated, objective, reproducible and reliable method to count HRF using an open-source software in DR. This allows, even without the contribution of an expert physician, to semi-automatically count HRF, which may represent an important biomarker of inflammation in DR. This will allow to identify and grade a specific phenotype of DR and DME and offer a contribute to clinicians in quantifying the change of this phenotype after treatment.

## Data Availability Statement

The raw data supporting the conclusions of this article will be made available by the authors, without undue reservation.

## Ethics Statement

The studies involving human participants were reviewed and approved by Ethics Committee for Clinical Practice of the Azienda Ospedaliera di Padova. The patients/participants provided their written informed consent to participate in this study.

## Author Contributions

Conceptualization, EM, LF, and EP. Methodology, TT, LF, RP, and EP. Validation, EP and EM. Formal analysis, TT, LF, RP, and EV. Investigation, TT, LF, EV, and EP. Resources, EP and EM. Data curation, TT, RP, and LF. Writing—original draft preparation, TT and EV. Writing—review and editing, LF, RP, and EM. Supervision, EM. All authors contributed to the article and approved the submitted version.

## Conflict of Interest

The authors declare that the research was conducted in the absence of any commercial or financial relationships that could be construed as a potential conflict of interest.
